# Publication trends in the medical informatics literature: 20 years of "Medical Informatics" in MeSH

**DOI:** 10.1186/1472-6947-9-7

**Published:** 2009-01-21

**Authors:** Jonathan P DeShazo, Donna L LaVallie, Fredric M Wolf

**Affiliations:** 1 Department of Medical Education and Biomedical Informatics, University of Washington, Box 357240, Seattle, WA 98195-7240, USA; 2 Department of Health Services, University of Washington, Box 357240, Seattle, WA 98195-7240, USA; 3 Department of Epidemiology, University of Washington, Box 357240, Seattle, WA 98195-7240, USA

## Abstract

**Background:**

The purpose of this study is to identify publication output, and research areas, as well as descriptively and quantitatively characterize the field of medical informatics through publication trend analysis over a twenty year period (1987–2006).

**Methods:**

A bibliometric analysis of medical informatics citations indexed in Medline was performed using publication trends, journal frequency, impact factors, MeSH term frequencies and characteristics of citations.

**Results:**

There were 77,023 medical informatics articles published during this 20 year period in 4,644 unique journals. The average annual article publication growth rate was 12%. The 50 identified medical informatics MeSH terms are rarely assigned together to the same document and are almost exclusively paired with a non-medical informatics MeSH term, suggesting a strong interdisciplinary trend. Trends in citations, journals, and MeSH categories of medical informatics output for the 20-year period are summarized. Average impact factor scores and weighted average impact factor scores increased over the 20-year period with two notable growth periods.

**Conclusion:**

There is a steadily growing presence and increasing visibility of medical informatics literature over the years. Patterns in research output that seem to characterize the historic trends and current components of the field of medical informatics suggest it may be a maturing discipline, and highlight specific journals in which the medical informatics literature appears most frequently, including general medical journals as well as informatics-specific journals.

## Background

Medical informatics has been emerging as a discipline over the past quarter century, along with the evolving, successive formal definitions that have been put forth, each one building on the previous [1]. The term "Medical Informatics" was introduced as a MeSH term in 1987. Previously known as "Information Systems", "Medical Informatics" is defined in MEDLINE as "The field of information science concerned with the analysis and dissemination of medical data through the application of computers to various aspects of health care and medicine."

In 1990, Greenes and Shortliffe described medical informatics as "the field that concerns itself with the cognitive, information processing, and communication tasks of medical practice, education, and research, including the information science and the technology to support these tasks."[2] Various other definitions of "medical informatics" exist and it appears the field continues to struggle with identity. Most descriptions and definitions of the field are consistent in pointing out the "multidisciplinary" and heterogeneous characteristics of the field. There is some disagreement with use of the term "medical" in reference to the field as "medical informatics" because the field encompasses all of healthcare, public health and biomedicine [3]. The rigor of scientific study in the field has also been a topic of focus, some questioning the dominant methodologies and where the field should locate within science, if at all [4-7]. Friedman has addressed this topic in the recent publication "Is medical informatics a mature science?" and concluded medical informatics may not yet be a mature discipline [8]. In the present study, we use the definition of medical informatics as operationalized by MEDLINE indexers: literature assigned a "Medical Informatics" MeSH designation will be considered medical informatics. We then survey the "evolution" of the field of medical informatics using bibliometric [9] and impact factor [10-12] analysis to help describe how the field has evolved, and in what directions.

Garfield and Sher [10,12] developed the impact factor in 1963 as a tool to quantitatively assess the relative frequency with which scientific articles are cited in subsequent publications. Journal impact factor represents the average number of times articles from the journal published in the past two years have been cited in the specified year of impact factor analysis. It is calculated by dividing current year citations to articles published in the previous two years, by total number of articles published in the previous two years [11,13].

Bilbiometrics has been defined as the use of statistical methods to analyze a body of literature to reveal historical development [14] and as the scientific and quantitative study of publications. Lewison and Devey [15] use the analogy that bibliometrics is to scientific papers as epidemiology is to patients. Because publication counts are a conventional metric of scientific output, bibliometric analysis have also been linked to funding and the financial bottom line of research[16]

Bibliometric studies date back to the early twentieth century and were furthered through the theoretical work of Derek de Solla Price [17,18] and practical work of Eugene Garfield [12,19]. This methodology has been utilized in multiple fields such as psychology, pharmacology, health education, pediatric dentistry, nursing informatics and others to describe the research and evolution of a discipline through output and citation analyses [20-26]. Within the medical informatics literature specifically, bibliometric studies have been used to characterize sub-domains and components of the field such as, modeling[27], computer-based medical records[28], and the medical informatics output of a country of origin[29,30]. Andrews used a co-citation analyses method to visualize scholarly communication in the field, as well as identify the most productive and prominent authors[31]. Studies have also used citation analysis to develop a core set of medical informatics serials [32-34]. A Morris and colleagues cocitation analysis found evidence of a maturing interdisciplinary field when it identified a relatively small core literature[34]. In contrast to Morris and colleague's study which only looked at the years 1993–1995, Synnestvedt and colleagues looked at the period from 1964–2004 to visually map highly cited authors [35,36]. Co-citation analysis is an important tool in bibliometric analysis, however analyzing publication output trends offers complementary information. For example, LaVallie and Wolf used publication counts and Impact Factor to descriptively characterize the field over a period of eight years[37].

Conventionally, the body of literature used in previous bibliometric analyses has been defined by either: 1) selecting a narrow body of literature (such as five core medical informatics journals), or 2) by searching numerous journals on a narrowly defined topic (such as "computer-based medical records". These approaches may not accurately reflect the complete body of medical informatics literature due to the evolving, multi-disciplinary nature of the field. Frequently new sub-domains appear and research is often published in non-medical informatics journals.

Biomedical researchers and scientists regularly consult sentinel journals, however they most frequently use PubMed when information seeking [38,39]. Defining medical informatics literature as articles assigned "Medical Informatics" MeSH (Major Topic) headings by National Library of Medicine indexers may result in a more comprehensive analysis and more practical results for researchers. This method of definition may more closely resemble how researchers actually search for and retrieve literature.

While a number of bibliometric studies have appeared in the medical informatics literature, none have attempted to describe the field as a whole throughout the defining time period of the last twenty years. Furthermore, researchers may benefit from a bibliometric analysis where the body of literature is defined in a manner accordant to their own information-seeking behaviors, which relies heavily on PubMed.

In the present study we describe trends in volume of medical informatics MEDLINE-indexed publications, identify major journals of publication and present trends in impact factor scores during the 1987–2006 period.

We address the following questions regarding the field of medical informatics:

1. What are the major research areas of medical informatics and how have they changed over the years?

2. To what degree is medical informatics more referenced in an application domain (e.g. as a component of a cardiology manuscript) versus medical informatics as an independent discipline?

3. In which specific journals can medical informatics literature be most found most frequently? Does Bradford's Law of scattering illustrate the core journals of the field and are those consistent with other groupings.

4. How does the publication output frequency in the field of medical informatics compare with other medical disciplines?

5. To what degree does the medical informatics literature exhibit linear or exponential growth as evidenced formally by Price's Law?

## Methods

To identify medical informatics publications, we searched MEDLINE/PubMed in March 2008 for all documents assigned the term "Medical Informatics" [MeSH terms] or any term in the "Medical Informatics" hierarchy as a Major Topic for each year of the 1987–2006 period. The specific search was "Medical Informatics" [MeSH terms] using the 'exploded' feature. Search results revealed 77,023 citations and included original articles, brief articles, reviews, editorials, proceedings, etc. For the purposes of this study these were considered 'Medical Informatics MeSH-indexed' (MI-MeSH) citations. The resulting citation data was loaded into a SQL Server relational database using PubMed's XML export function and a freely available MEDLINE XML parser [40].

To estimate the growth of MI-MeSH literature, the annual growth rate for the sample period was compared with all of PubMed, as well as with MeSH categories: "Public Health", "Medicine", and "Surgery". "Public Health", "Medicine" and "Surgery" (all MeSH Major Topic) were selected as well known reference domains. The formula used for annual growth rate(AGR) = (Current Year Total – Previous Year Total)/Previous Year Total.

To assess whether medical informatics follows Price's Law of exponential growth [17,18], we fit the number of MI-MeSH citations per year for the study period to a linear equation as well as an exponential curve. These two equations are y= 356.32 x-707575.22 (*R*^2 ^= .79) and y = 94.19*exp(.2236*x-444.03)+1798.61 (*R*^2 ^= .97) respectively.

Fifty MI-MeSH terms are included in the PubMed MeSH browser. These terms are considered in 2006 to be under the "Medical Informatics" MESH hierarchy. The presence of one or more of these MI-MeSH terms assigned to a document as a Major Topic indicates it is a "Medical Informatics" indexed document. All citations in the study corpus are identified by at least one MI-MeSH term, based on the initial selection criteria, plus up to thirty additional MeSH headings (the maximum found is 26 concurrent Major Topics). We calculated the annual citation frequency for each of these terms, as well as the frequency of which MI-MeSH terms appear together. A principal components analysis was performed to examine whether certain MI-MeSH terms tend to be used and cluster together in any discernable pattern across this 20-year time period.

To evaluate the trends in journals, we apply Bradford's law of scattering[41] to all journals publishing medical informatics articles and also compare 'core' journal sets from various sources [41]. Individual journals publishing 20 or more articles per year for each of the twenty years were identified. These are labeled 20+MI, which we considered an index of the top MI-MeSH Journals. This group of journal titles is compared with those found utilizing another method of identifying medical informatics journals: the ISI Journal Citation Reports journals categorized as "Medical Informatics". The Journal Citation Reports provides a list of journals based on Subject Category of "Medical Informatics"[42]. These Journals are considered the JCR MI Journals.

We searched ISI Journal Citation Reports to identify journal impact factor ratings for each journal publishing twenty or more MI-MeSH articles (20+MI Journals) for each year during the period. Impact factor journal ratings were available for many 20+MI journals for all years from 1987 through 2006. An average impact score per year was calculated by dividing the sum of the impact factors by the number of journals. A weighted average impact score was calculated by adjusting each impact factor for the relative number of MI-MeSH citations for that year (the sum of journal impact factors multiplied by the percentage of citations attributable to that journal). The impact factor is not intended for use as a means of critiquing journals in terms of inter-journal quality assessment. We discuss this further in limitations.

## Results and discussion

### Publication Trends of Medical Informatics

The total number of medical informatics MeSH-indexed (MI-MeSH) articles retrieved for the 20-year period was 77,023. In 1987 and 2006 there were 1,272 and 9,973 MI-MeSH articles, respectively. This indicates a 784% growth in annual citations over the twenty-year time period.

**Figure 1:**
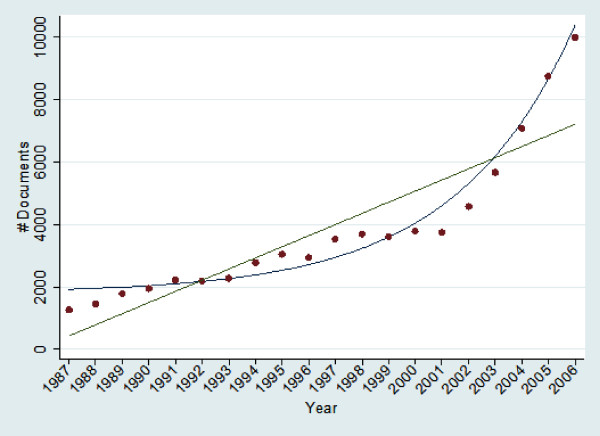
**Growth in the output of medical informatics**. We fit the number of MI-MeSH citations per year for the study period to a linear equation as well as an exponential curve. These two equations are y = 356.32 x-707575.22 (*R*^2 ^= .79) and y = 94.19*exp(.2236*x-444.03)+1798.61 (*R*^2 ^= .97) respectively.

**Figure 2:**
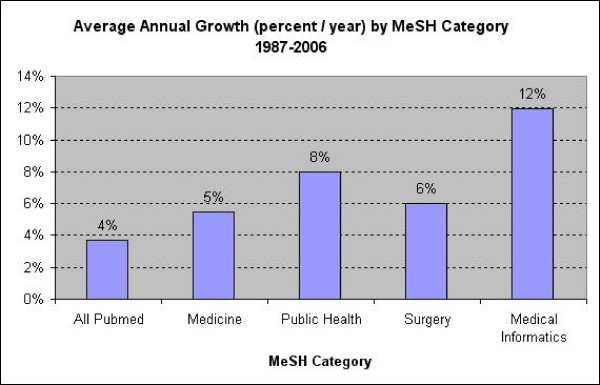
**Average annual growth rates**. Comparison of average annual growth rates among Medical Informatics and reference MeSH categories

Medical informatics indexed articles grew by an average of 12% each year over the study period, and appeared to fit an offset exponential growth curve rather than a linear equation. An exponential curve explains 97% of the variance in medical informatics citations over this 20 year period, while a linear equation explains just 79%. (See Figure 1) While these data are not compelling on their own, they support the postulates of Prices Law. To compare medical informatics growth rate with well known reference growth rates, Figure 2 and Table 1 illustrate the 5-year average and overall average growth rate of PubMed, "Public Health", "Medicine", and "Surgery", as well as "Medical Informatics".

**Table 1:** Growth of Medical Informatics.

Period	All PubMed	Public Health	Medicine	Surgery	Medical Informatics
**1987–1991**	43927 (12%)	5724 (48%)	1768 (29%)	135 (48%)	773 (66%)

**1992–1996**	37981 (9%)	2681 (15%)	1966 (25%)	44 (11%)	714 (38%)

**1997–2001**	145868 (37%)	12888 (68%)	3339 (36%)	226 (50%)	229 (7%)

**2002–2006**	102423 (17%)	10882 (28%)	1722 (12%)	132 (23%)	4949(120%)

Average annual growth (Number of articles) and percentage growth over 5-Year periods from 1987–2006

The total number of unique journals over this twenty year period is 4,655. The vast majority of journals contain relatively few MI-MeSH articles. For example, 81% of the journals average only one MI-MeSH citation per year or fewer.

We applied Bradford's Law of scattering and divided the output frequency ranked journals into three groups, with each group of journals representing approximately the same number of articles. For example, the highest MI-MeSH output journals wouold be in the first group, and the lowest would be in the last group. Only 35 journals were needed to represent one third (25,661) of the total 77,023 published MI-MeSH articles. In contrast, 286 and 4,323 journals made up the next two thirds, respectively (See Table 2).

**Table 2:** Distribution of journals number of journals by grouping articles into one thirds.

	*Journals*		*Articles*		*Cumulative Total*
**Group**	**No.**	**%**	**No.**	**%**	

Top 3^rd^	35	0.7	25661	33.3	25661

Middle 3^rd^	286	6.2	25656	33.3	51517

Bottom 3^rd^	4323	93.1	25706	33.4	77023

Total	4644	100	77,023	100	77,023

**Figure 3:**
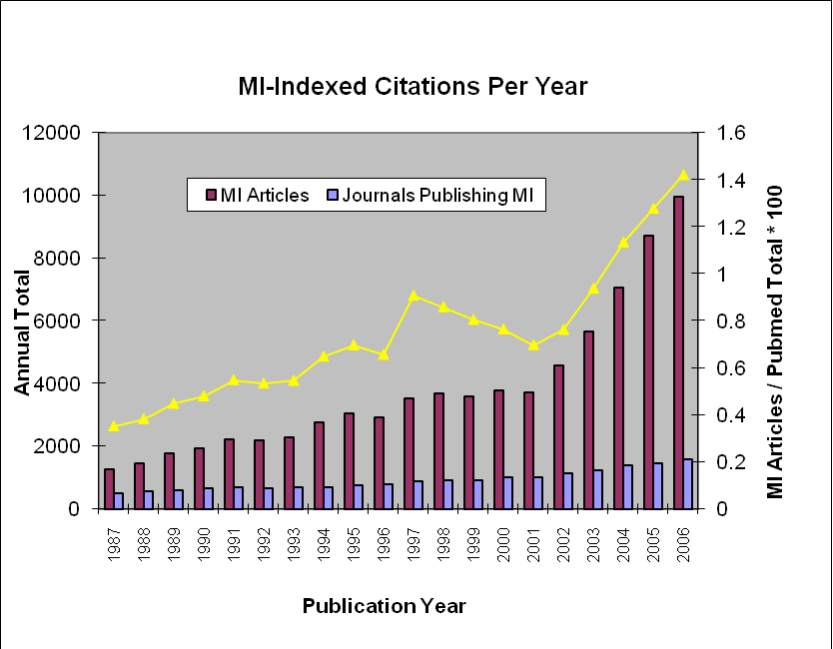
**Trends in medical informatics publication output over twenty years by number of articles and number of discrete journals**.

Growth in the number of journals publishing MI-MeSH articles increased over the 20 year period from 500 to 1578, however this growth curve was more conservative than the number of articles. (See Figure 3)

The journals with the greatest numbers of MI-MeSH publications, as well as the ISI Journal Citation Reports (JCR) MI journals, are presented in Table 3. The ISI does not rank its journal list; however, we present the ISI journals ordered by 2006 MI-MeSH citation count for comparison with the MI-MeSH indexed list. The top five ISI Journals by 2006 MI-MeSH citation count are *IEEE Tran. on I.T. in Biomedicine*, *JAMIA*,*International Journal of Medical Informatics*, *Methods of Information in Medicine*, and *Biomedizinische Technik*. The *Journal of Biomedical Informatics *is found sixth in the ordered list. In contrast, seven of the 10 most indexed journals in the MI-MeSH list were imaging, physics, or engineering oriented, and two were conference proceedings.

**Table 3:** JCR subject MI vs. MI-MeSH.

Top MI-MeSH Indexed Journals Ranked by 2006 MI Output	'06 Pub Cnt.	JCR Subject Category Medical Informatics	'06 Pub Cnt.
Proc. IEEE Eng. in Med. and Biology Soc. Con.	390	*IEEE Tran. on I.T in Biomedicine	77
IEEE trans on image processing	326	*JAMIA	72
Medical physics	274	*International journal of medical informatics	55
Proc. AMIA Annual Symposium	271	Methods of information in medicine	49
Stud. in Health Technology & Info	270	Biomedizinische Technik	28
Physics in Med. and biology	227	Journal of biomedical informatics	24
Med. image Comp. & comp-ass inter.	224	IEEE engineering in medicine and biology magazine	26
IEEE transactions on bio-medical eng.	185	Computer methods and programs in biomedicine	22
Nucleic acids research	180	Computers, informatics, nursing: CIN	18
IEEE trans on pattern analysis and mach. Intel.	173	Artificial intelligence in medicine	16
BMC bioinformatics	150	Medical & Biological Engineering & Computing	9
Int. J. of radiation, onc, bio, phys	140	Medical Informatics and the Internet in Medicine	7
Bioinformatics (Oxford, England)	138	Inter. J. of Tech. Ass. in healthcare	4
IEEE trans. on medical imaging	135	Statistical Methods in Medical Research	3
Magnetic resonance in medicine	104	Journal of Evaluation in Clinical Practice	2
IEEE trans on visualization and comp graph	92	Med Decision Making	1
Healthcare informatics	91	Statistics in Medicine	1
Radiotherapy and Oncology	87	Journal of Cancer Education	1
Applied Optics	85		
J. biomedical optics	85		
IEEE trans on ultrason., ferro., and freqy contro	78		
Journal of AHIMA	78		
*IEEE trans on IT in biomedicine	77		
J of the American Society of Echocardy	74		
*JAMIA	72		
IEEE transactions on neural networks	65		
Modern healthcare	64		
Academic radiology	62		
Ultrasonics	61		
Optics letters	60		
Journal of magnetic resonance imaging: JMR	57		
Health management technology	56		
Medical image analysis	56		
J. Optical Society. Of America	55		
*Int J Medical Informatics	55		
J Healthcare Information Management	50		

A summary and comparison for the year 2006 of the number of medical informatics citations in ISI Journal Citation Reports (JCR) and MeSH (> 50 citations only). *Note top three JCR journals in MI-MeSH ranked list.

Journals publishing 20 or more MI-MeSH indexed articles per year were identified for each of the given years. In 1987 there were 6 such journals: *Computers in Healthcare*; *Healthcare Computing and Communications*; *Hospitals*; *The Journal of Medical Systems*; *Frontiers of Radiation Therapy and Oncology*; and *The Health Services Journal*. The number of MI-MeSH Journals with > 20 publications per year increased steadily to 23 in the year 2000, and by 2006 had reached 77 journals.

### MeSH Terms in MI-MeSH

**Figure 4:**
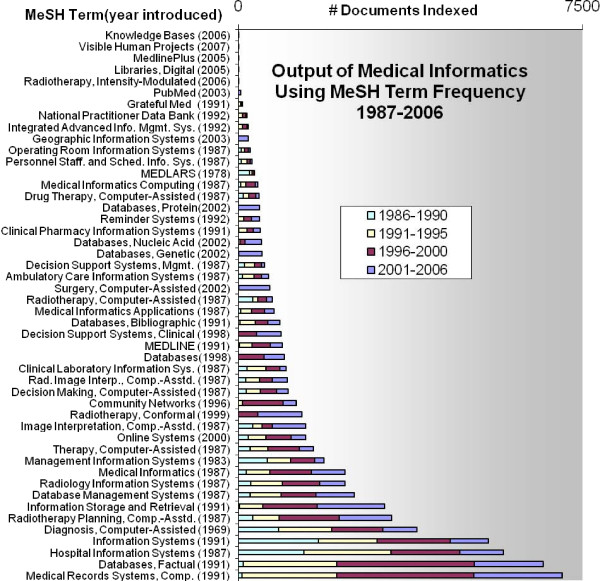
**2007 MI MeSH (and year introduced)**. Terms and their publication frequency for the period 1987–2006 summarized by 5-year intervals.

We found that each MI-MeSH article in the study corpus was assigned between 1 and 27 MeSH headings as Major Topics. There are currently 50 MI-MeSH terms used in MEDLINE/Pubmed which are identified by their position under the "Medical Informatics" MeSH Tree Hierarchy. Figure 4 lists the 50 MI-MeSH headings as well as their frequency assigned as Major Topic over the 20 year period.

The majority of the study sample of citations had central concepts outside of medical informatics as indicated by being assigned non-MI MeSH Major Topics. 54,160 (70%) citations found in the MI-MeSH collection were also indexed by a non-MI MeSH term (Major Topic). It was also very rare that a citation was indexed as being only medical informatics. 76,119 (99%) of the citations were also assigned MeSH terms(Major or Minor Topic) that were not related to medical informatics. An analysis by year indicated this interdisciplinary trend was relatively consistent over the years.

MI-MeSH terms, also known as designators, were rarely assigned together to the same documents (see Table 3). Table 3 summarizes the frequency with which MI-MeSH terms are assigned together on individual manuscripts for the years 1996 and 2001. For the entire study period, approximately 73% of the documents were assigned only one MI-MeSH (Major or Minor Topic) designator. Looking at Major Topic MI-MeSH designators, only 5,244 (7%) of the documents were concurrently assigned two or more Major Topic MI-MeSH terms. No grouping patterns between MI-MeSH terms could be found. The principal components analysis indicated MI-MeSH terms were being assigned to documents almost mutually exclusive of one another. This trend appears to be consistent over the 20 year period.

### Impact Factor Results

**Figure 5:**
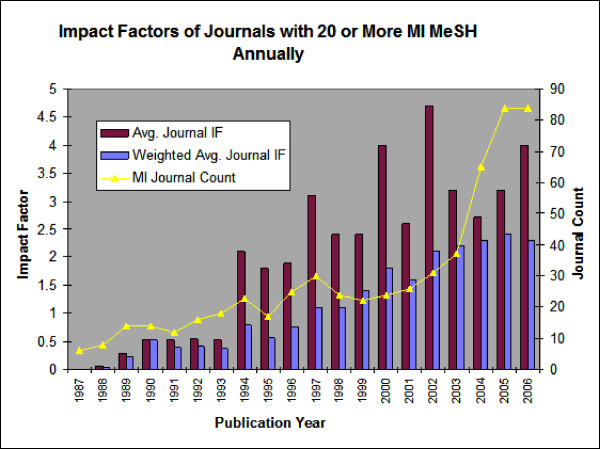
**Impact factors of journals with 20 or more MI-MeSH annually**.

Impact factors were identified for all Journals that published 20 or more MI-MeSH (20+MI) indexed articles in a year. In 1987, there were 6 journals with 20+MI articles, yet none had an impact factor for that year. In 1988, there were 8 journals and one had an impact factor: *Computer Methods and Programs in Biomedicine*. The number of 20+MI journals increased steadily, as did the impact factors during the 20-year period, marked by steady growth in the final 12 years. (see Figure 5) By 2006, 111 journals carried 20+MI articles, and 82 were assigned impact factors.

Average impact score and weighted average impact score show little increase in the first two years, then a notable jump with little increase between 1988–1993, and steady increase from 1993–2006. The unweighted average impact score showed marked spikes in 1994, 1997, 2000, and 2002 which are not reflected in the weighted average. These increases may be explained by the following data. By 1989, almost half of the journals had impact factors, yet they remained relatively low values. By 1994, journals with comparably higher impact factors (such as *Nucleic Acids Research *and *Human Molecular Genetics*) were appearing and remaining on the 20+MI journal list in subsequent years. A look at the spikes in the unweighted average reveals the first appearance and dominating effect of 'very high' impact factor journals such as *Nature *and *Science*. Journals such as these intermittently publish greater than twenty MI-MeSH articles in a year, thus creating the spikes in the un-weighted impact factor value.

In the current environment, information technology (IT) is being cited as "high priority for the American health care system and the U.S. economy. IT is a pivotal part of transforming our health care system," as stated in a Health and Human Services Health Information Technology Leadership Panel report [43]. Current study findings are consistent with the "maturation" of evaluation studies in medical informatics research and "strong shift" from medical journals to medical informatics journals reported by Ammenwerth and de Keizer in their review addressing trends in evaluation studies of IT in health care [44].

Using publication data, we have described trends in the volume and subject areas of medical informatics research output for the 1987–2006 period and provided insight into growth and increasing "visibility" of the field through assignment of yearly total impact scores.

### Medical Informatics as multidisciplinary and interdisciplinary

Descriptive evidence of the multidisciplinary and interdisciplinary nature of medical informatics is detectable using the journal classification system in place at PubMed and the National Library of Medicine as a metric. Previous studies using similar methods as well as co-citation analysis support this as well[32,37,45]. However, this study found it difficult to enumerate these concepts based on our methods and data especially without comparison to other disciplines. Virtually no research output was exclusively and solely indexed as medical informatics. The analysis of MeSH terms assigned to the same document indicated that MI-MeSH terms were almost exclusively indexed along side non-medical informatics designators. In addition, roughly 26% of MI-MeSH terms were assigned along side another MI-MeSH terms (Major or Minor Topic) and fewer than 7% were assigned with another MI-MeSH Major Topic. No clusters of medical informatics-specific topics could be detected through principal components analysis. Non-medical informatics MeSH were often indexed as concurrent major headings, indicating the article is at least as relevant to the domain area as it is to medical informatics. Furthermore, we see the journals which publish an overwhelming majority of medical informatics indexed literature are not typically identified as medical informatics-specific journals. Multidisciplinary fields require a multitude of terms for MeSH indexing, however the majority of MI-MeSH terms are specified technologies (such as a protein database and medical record system) while very few are concepts (such as computer assisted therapy).

Using MeSH to indentify core concepts within a discipline is problematic at best. However it should be no less problematic in theory to use MeSH terms than other indexing terms such as JCR subject categories which have previously been used successfully in bibliometric analysis[45]. MeSH terms are much more detailed than JCR subjects categories and possess inherent hierarchical relationships which may glean additional insight.

From a MeSH perspective, medical informatics could be described as a set of largely mutually exclusive tools or concepts which are most frequently applied to address problems in specific applied domains and in non medical informatics contexts.

### Medical Informatics as a Mature Discipline

The rapidly accelerating growth rate of the discipline may indicate a high rate of change and be consistent with the maturation process. The growth demonstrated by this data is consistent with Prices Law and may indicate a typical scientific field. As a field, the cumulative output for medical informatics is increasing at a rate higher than comparison fields. There is a notable increase in the productivity of field beginning 2001. One possible explanation for this increase may be the result of structural changes within the field just prior to and during this time period. For example, funding for both categorical research (e.g. bioterrorism) and training (e.g. NLM Training Grants) was becoming available during this time period and medical informatics departments, divisions, or units were forming in universities.

Some MI-MeSH terms were more prevalent early in the 20-year period and utilizations of some appear to be on a relative decline in publication rate during the study period. A closer look at specific areas within medical informatics, as represented by MeSH terms, indicates possible increasing maturity and subsequent decline in output. For example, Radio Therapy Planning, Computer Assisted MI-MeSH term saw a significant increase in citations in the 1997–2001 and 2002–2006 periods when compared with earlier periods. Also, Community Networks MI-MeSH (introduced in 1996) saw a significant decline in output in the 2002–2006 period. However, MI-MeSH terms are time period specific and often belatedly reflect trends in the field. This reaction phenomenon may give the illusion of a spike and subsequent regression. Furthermore, the renaming or relocation of a MeSH term in the hierarchy may also give the illusion of a decline in a specific area. Conceptually, a new field of basic science may have tumultuous and significant growth in research output before leveling off to a more consistent output. However, an applied field's research output may never level off if it is successful at continuously identifying new application domain areas to explore, in addition to being subsequently fueled by useful scientific innovations from its foundation disciplines.

The scattering of medical informatics literature may also indicate a maturing field. Bradford's Law suggests the presence of a core set of journals which can be identified by analyzing the scattering may indicate a mature discipline. In medical informatics this core set of journals represents less than one percent of the total number of journals, which is a relatively tight cluster.

### Finding Medical Informatics Literature

Although a third of the medical informatics output can be found in less than 1% of the journals, there are still more than 100 different journals which publish more than 20 MI-MeSH articles per year. However, the bulk may be found in journals not generally identified as medical informatics specific journals. There are also various interpretations of what journals should be considered medical informatics. Highly visible journals such as *JAMIA*, *Journal of Biomedical Informatics*, and *Medical Decision Making *are likely to be considered by many in the field as the most significant medical informatics journals. However the Journal Citation Report "Medical Informatics" (JCR MI) category journals are found in general low on the output rank ordered list. In fact, several of the JCR MI Category journals have fewer than 10 MI-MeSH (Major Topic) indexed articles in 2006.

Our findings differ from previous studies that identified no more than 30 publications related to medical informatics[30,34]. Our 'core' set of medical informatics journals differs predominately by the inclusion of more engineering, physics, and imaging journals in our top ranked list. This may be in part due to our straightforward, bottom-up strategy of including any journal by simply ranking on frequency of publishing any MI-MeSH assigned article. In contrast, previous studies limited the initial corpus of journals either by manually searching indexes by the explicit keyword "medical informatics", by subjective selection, or a combination thereof. For example, Morris and colleague's derived 20 core medical informatics journals from analyzing an initial corpus of 29 journals that were selected based on keyword searches and some subjection [45].

When looking for medical informatics literature, it is important to recognize the possibility of biases and inconsistencies in journal classification as well as very large dispersion of medical informatics research throughout the literature. It could be that articles in medical informatics research are more closely aligned with the application domain journals rather than medical informatics specific journals. However, it is more likely the conventional or common consensus list of 'medical informatics journals' is outdated. Medical engineering, physics, optics, and imaging journals consistently publish large numbers of articles which are indexed as MI-MeSH, yet these journals are not generally identified as 'medical informatics' by the medical informatics field as JCR MI groupings.

### Limitations

We acknowledge the following limitations: Our search strategy only employed the "Medical Informatics" MeSH term and its descendents in the MeSH hierarchy. We did not attempt to answer which articles are 'truly' medical informatics. We envisioned the more general MeSH term "Medical Informatics" as an umbrella term through which we anticipated capturing a large volume of the indexed literature in this field (as opposed to countless other specific terms utilized for indexing of known medical informatics articles). The research output quantity described does not represent the entire medical informatics research output; but acts as a proxy to provide data for a historical overview of trends. For example, "Bioinformatics" and "Public Health Informatics" are independent MeSH subjects not under the "Medical Informatics" term hierarchy and consequently are not included in this study. The MeSH term "Informatics" was introduced in 2005 as an umbrella category for "Dental Informatics", "Medical Informatics", Nursing Informatics" and "Public Heath Informatics". The overarching themes between these subdomains are thought to be significant and related. Along these lines, a more complete study may have included the entire "Informatics" hierarchy. Similarly, studies such as this are most useful when current are quickly outdated. However, we feel this study adequately illustrates and important event: the first twenty years of the use of "Medical Informatics" in MeSH.

Furthermore, the implications of using MeSH assignments to define the body of literature and journals may result in ignoring citation patterns and temporal incongruence. For example, the appearance of new MeSH terms is largely reactive and tends to somewhat belatedly follow trends in research output. While new terms may be assigned to literature retrospectively, MeSH trends may be chronologically behind actual trends. Defining literature through citation analysis may also give a more accurate representation of the contributors (both article and journal) to the knowledge domain.

There are several bibliometric measures which were not examined for this study, which may have provided valuable information to this analysis. This study focuses on journal and article as the primary unit of interests, and therefore did not examine citation or author centered bibliometric indicators. Another useful bibliometric measure not looked at in this study is the National Participation Index (PI), which illustrates the relative contribution to the field by country of origin[26].

The indexing of an article by one or more MeSH terms is ultimately subjective, although indexers are highly trained subject matter experts and follow explicit indexing procedures. As previously noted, we were interested in describing historical category trends and output venues as opposed to quantitatively determining actual output for the period of study. Limitations of the use of impact factors in this study include first, that only original research and review articles are counted as published articles; however, citation counts include original research, reviews plus letters, editorials and new items. Second, a change in journal format/size year to year may result in temporary IF increases or decreases. Third, journal title changes will result in a theoretic loss of citations, as the IF is calculated utilizing articles published by that journal over the previous two years.

Lastly, MeSH is updated annually and may change over the years. Terms may be introduced, removed, and also relocated to other positions in the MeSH hierarchy during the study period. Using the 2006 MI-MeSH terms for this study describes the current consensus of "Medical Informatics", however it disregards any MeSH terms which were previously considered medical informatics but removed from the "Medical Informatics" hierarchy prior to 2006. For example, "Emergency Medical Service Communication Systems" was indexed under the "Medical Informatics" hierarchy prior to 1991, when it was renamed to "Emergency Care Information Systems". "Emergency Medical Service Communication Systems" is currently is located under "Emergency Medical Services".

## Conclusion

By describing the literature using MeSH, this bibliometric analysis captured a significant number of articles assigned to "Medical Informatics" by NLM indexers irrespective of the journal's core domain. For example, articles may be assigned to "Medical Informatics" even when "informatics" is not explicitly in the journal title. This approach has the potential to significantly increase the sensitivity of the analysis by adding a human curated nature to the classification, and capturing the numerous literature published in non-medical informatics specific journals. Furthermore, our methods of classification are somewhat aligned with the methods researchers use to find literature themselves; therefore the results of the analysis may in turn be more useful than one which in which the body of work is defined by other means, such as by key journal. Another significant consequence of using MeSH extensively in article classification is that the study results may reflect as much on the use of medical informatics terms in MeSH as they are a reflection of the medical informatics field as a whole. In other words, the results are a description of the field as told through the vocabulary of MeSH, which is designed for other purposes.

There has been a steady growing presence of medical informatics articles and journals in the published literature discipline over time. Impact factors patterns may also reflect the increasing visibility, breath and attention to this rapidly evolving field. However, impact factors likely have limitations as well, such as the effect of biases and quality misconceptions. For example, not all articles in a high impact factor journal have equivalent quality and certain types. This analysis does suggest that output in the medical informatics field is primarily growing across three dimensions: 1) The number of medical informatics journals, 2) The overall number of medical informatics indexed articles, and 3) The fact that medical informatics indexed articles are appearing more frequently in non-medical informatics journals, most notably in some journals of very high quality and visibility (as measured by impact factor).

Results of our study note the specific journals of publication of medical informatics research and complementary disciplines, including core medical informatics journals and journals which may be lesser known. By highlighting medical informatics literature output trends, this may serve as a useful guide for researchers looking for medical informatics literature or appropriate publication venues.

Lastly, this study also highlights the need for refinement in utilization of the "Medical Informatics" term for MeSH indexing. This is supported by the finding of the low MI-MeSH index rate of articles published in core medical informatics journals, indicating that many articles published in core medical informatics journals are not classified as under "Medical Informatics" in MeSH. MI-MeSH indexing patterns such as the apparent mutual exclusivity of MI-MeSH terms, and the concordance with application domain terms may also suggest incongruence between the assignment of MeSH terms and conventional thought regarding the field's taxonomy. Further study of individual articles within medical informatics-dedicated journals to assess overall output subject trends might well contribute to our understanding of the trends and impact of the field as a whole.

## Authors' contributions

JD participated in the study design, performed the data collection and analysis, and helped to draft the manuscript. DL conceived of the study, participated in its design and coordination and helped to draft the manuscript. FW participated in the study design and coordination and helped draft the manuscript. All authors read and approved the final manuscript.

## Pre-publication history

The pre-publication history for this paper can be accessed here:

http://www.biomedcentral.com/1472-6947/9/7/prepub
